# Updated Advances on Drugs and Bone-Targeting Nanoparticles for Osteoporosis Therapy: Carrier Materials, Modification, Function Mechanism, and Applications—A Systematic Review

**DOI:** 10.3390/ph18121809

**Published:** 2025-11-27

**Authors:** Yehao Lin, Yidong Xu, Siyue Zhou, Junyu Liu, Min Zhang, Baoxin Zhang, Haixia Chen

**Affiliations:** 1 Tianjin Key Laboratory for Modern Drug Delivery & High-Efficiency, School of Pharmaceutical Science and Technology, Faculty of Medicine, Tianjin University, Tianjin 300072, China; linyehao@tju.edu.cn (Y.L.); mita@tju.edu.cn (Y.X.); zhou_624@tju.edu.cn (S.Z.); junyuliu@tju.edu.cn (J.L.); 2 Nutritious and Healthy Food SinoThailand Joint Research Center, College of Food Science and Biological Engineering, Tianjin Agricultural University, Tianjin 300384, China; 3 State Key Laboratory of Nutrition and Safety, Tianjin University of Science & Technology, Tianjin 300457, China; 4 The Second Affiliated Hospital of Inner Mongolia Medical University, Hohhot 010050, China; z197218275@yeah.net

**Keywords:** osteoporosis therapy, bone-targeting nanoparticles: carrier materials, modification, function mechanism

## Abstract

**Background:** Osteoporosis is one of the most common bone metabolic diseases that affects mainly the health of elderly people. It is a kind of prevalent chronic disease, and the conventional treatment methods have some limitations or side effects. Targeting nanoparticles represent a novel technology that has garnered extensive attention in recent years. They can selectively enhance the drug concentration at the targeted site, offering a novel treatment method. **Methods:** The review is carried out according to the Preferred Reporting Items for Systematic Reviews (PRISMA 2020) guidelines. **Results:** This article comprehensively summarizes recent research progress on the status of existing anti-osteoporosis drugs and bone-targeting nanoparticles for the treatment of osteoporosis, including their carrier materials, modification techniques, preparation methods, and function mechanisms. It also discusses their applications in RNA interference (RNAi) therapy and other related areas. Furthermore, given the limitations of bone-targeting nanoparticles, solutions and viewpoints have been proposed. This review summarizes that bone-targeting nanoparticles are useful for osteoporosis therapy and provide a novel perspective for new drug discovery. **Conclusions:** Bone-targeting nanoparticles overcome the limitations of traditional treatment methods and enhance therapeutic efficacy. However, the clinical translation of bone-targeted nanoparticles remains challenging and requires further investigation.

## 1. Introduction

Osteoporosis is an osteolytic disease characterized by low bone mass and deterioration of bone microstructure, with a high incidence worldwide [1]. This condition significantly increases a patient’s risk of fractures due to osteoporosis, with an average of one person suffering a fracture every three seconds globally. This phenomenon is growing, and it is predicted that the number of global fracture patients will rise by 50% by 2030. The primary pathological feature of osteoporosis is the disruption of the balance between osteoblast-mediated bone formation and osteoclast-mediated bone resorption, with decreased osteoblast activity and increased osteoclast activity [2]. The incidence of osteoporosis varies among different races and age groups. Compared with individuals in Europe and America, the incidence rate is higher among Asians. Middle-aged and elderly individuals are more susceptible to osteoporosis [3]. Both age and menopause can contribute to osteoporosis. The diagnosis of osteoporosis is often based on bone mineral density measurements. Unfortunately, although osteoporosis poses a significant threat to public health, most patients do not receive timely treatment due to its inconspicuous early characteristics [4]. Patients at high risk of fractures actually benefit less. The treatment of osteoporosis requires significant human and financial resources. In the United States, the average cost of treating osteoporotic fractures per patient ranges from several thousand to tens of thousands of dollars. Currently, there are over 90 million osteoporosis patients in China. By the middle of this century, this number is expected to exceed 100 million, and China’s expenditure on the treatment of osteoporotic fractures will exceed 100 billion dollars [5], imposing a significant burden on both patients and society. Currently, most methods of drug administration for the treatment of osteoporosis involve systemic delivery, which can lead to certain side effects and result in poor patient compliance [6]. The development of new methods for treating osteoporosis is a crucial endeavor in the field of medicine.

Nanoparticle is an innovative drug delivery system characterized by exceptional biocompatibility and stability. After years of development, nanoparticle technology has undergone significant improvements. Bone-targeting nanoparticles achieve specific binding to bones through targeted ligands, resulting in selective accumulation at bone sites, leading to substantial breakthroughs in enhancing drug targeting and minimizing toxic side effects. In the medical field, nanoparticles have been applied to the treatment of bone diseases, effectively addressing the limitations of traditional treatment methods. This approach offers a novel solution for managing bone diseases such as osteoporosis.

Though there are several reviews on osteoporosis [7,8,9], which is focused on the limited aspects. The newly applied materials, preparation methods, and action mechanisms of nanoparticles in recent years are rarely systematically summarized. In this review, we systematically elaborate on the above contents, the progress on drugs and introduce advanced nanoparticles for RNA interference (RNAi) therapy on osteoporosis. Considering the advantages and limitations of bone-targeting nanoparticles, this review aims to provide researchers with insights into their potential and value in the treatment of osteoporosis.

## 2. Materials and Methods

This systematic review was conducted in accordance with the Guidelines for Review and Meta-Analysis (PRISMA) statement [10] The completed PRISMA checklist is provided in Supplementary Materials. The protocol was registered in the PROSPERO database (CRD420251176418).

### 2.1. Eligibility Criteria

This review summarizes the advances in drugs and bone-targeting nanoparticles. The search included only articles and patents in English, published in the past 10 years (2016–2025), and containing the selected keywords.

### 2.2. Information Sources

Information for the past 10 years was searched using the databases PubMed and Web of Science, and the review was carried out according to the Preferred Reporting Items for Systematic Reviews (PRISMA 2020) guidelines [10].

### 2.3. Search Strategy

The primary drugs and bone-targeting nanoparticles for treating osteoporosis are listed based on the literature, and different categories of drug delivery systems were used as terms to retrieve related introductions (Table 1).

### 2.4. Study Selection and Data Collection Process

We used the PRISMA 2020 flowchart to organize key data, with titles and abstracts independently screened and evaluated by different reviewers. This article only includes the results obtained from the Web of Science and PubMed databases and excludes non-English articles. Essential information for each study, such as the title, author, and journal of publication, was recorded in a Microsoft Excel spreadsheet. Duplicate items were removed and then filtered. Any discrepancies in the reviewers’ judgments were resolved through discussion until a consensus was reached.

## 3. Results and Discussion

### 3.1. Database Search and Included Studies

**Figure 1:**
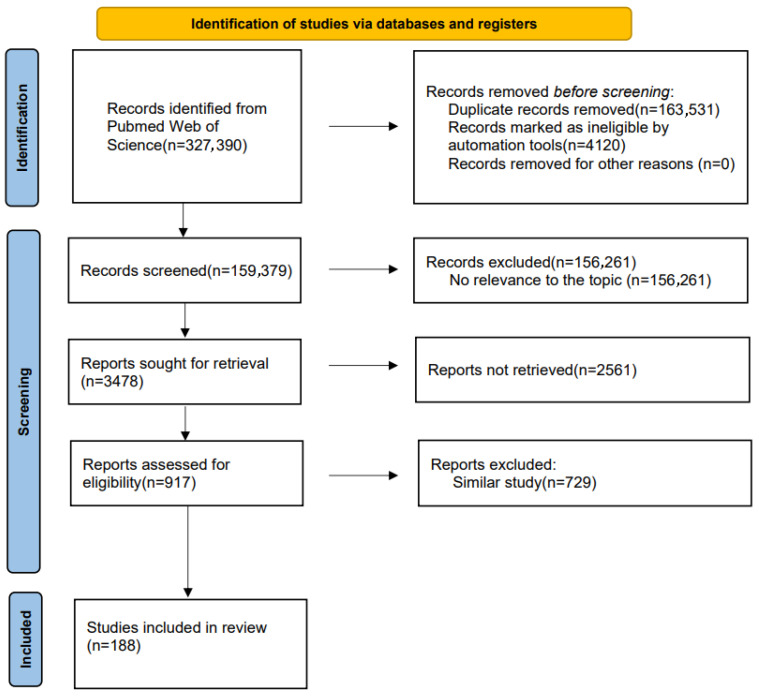
The flowchart of the selection process of literature and reports based on PRISMA.

The flowchart illustrating the literature retrieval and selection process for this review is presented in Figure 1. Two authors retrieved the research. In cases of inconsistent answers, following the discussion results and joint decision, a total of 327,390 records were initially identified. Of these, 167,651 records were excluded for various reasons: conference papers, book chapters, news articles, etc. Following a database screening that eliminated duplicate entries and records not pertinent to the topic, 3478 studies were ultimately identified. Subsequent screening of titles and abstracts resulted in the retention of 917 records due to their relevance and alignment with the scope of this review. The full texts of these papers were then thoroughly reviewed and assessed to ascertain their compliance with the criteria established for this scoping review. Ultimately, 188 studies were included in the final analysis. 

### 3.2. The Pathogenesis of Osteoporosis

From the perspective of pathogenesis, osteoporosis can be classified into primary and secondary osteoporosis. Primary osteoporosis is further categorized into postmenopausal osteoporosis, senile osteoporosis, and idiopathic osteoporosis [11,12]. Among these, postmenopausal osteoporosis is a more representative one, and a prevalent form, with postmenopausal women constituting an important group of osteoporosis patients [13,14]. After menopause, the concentrations of sex hormones in women’s bodies decline, disrupting bone homeostasis and subsequently triggering a series of reactions, including osteoporosis. Estrogen plays a crucial protective role in bone health by affecting osteoblasts, osteoclasts, and mesenchymal stem cells, thereby significantly safeguarding bone integrity [15,16,17]. Studies have shown that estrogen promotes autophagy, which facilitates the differentiation of osteoblasts [18], effectively prevents their apoptosis, and thereby extends their lifespan. When estrogen secretion is insufficient, there is an increase in osteoclast activity. After menopause, the body’s secretion of estrogen is significantly reduced, making individuals more susceptible to osteoporosis. Senile osteoporosis is primarily caused by aging. As the body ages, all physiological functions decline, leading to an increase in the number of senescent cells. This cellular aging contributes to the development of osteoporosis. The pathogenesis of senile osteoporosis is primarily associated with osteogenic defects, diminished osteoblast function, insufficient bone formation, and eventual bone loss. These changes can adversely affect bone health and are significant contributors to important factors in inducing senile osteoporosis. Other types of osteoporosis with unknown causes are called idiopathic osteoporosis. Among adolescent patients, some can resolve themselves, but some will suffer severe damage. Genetic factors may be one of the causes of specific osteoporosis [19]. Osteoporosis will need different therapy methods based on the different pathogenesis.

Secondary osteoporosis is a condition that arises from underlying diseases or the use of certain medications. Bone disease is a significant complication associated with diabetes. Compared to type Ⅱ diabetes, type Ⅰ diabetes is associated with a significant incidence of osteoporosis [20], and the research on this topic is relatively comprehensive. Generally, diabetes contributes to osteoporosis through various mechanisms, including interference with metabolic processes and the effects of reactive oxygen species and inflammatory molecules [21,22]. Diabetic patients often exhibit elevated urinary permeability, leading to the loss of these trace elements, such as calcium, in the urine. This loss can disrupt the normal levels of trace elements in the body, ultimately resulting in metabolic disorders [23]. In addition, hyperglycemia results in an elevated level of the secretion of ROS, advanced glycation end-products, and inflammatory cytokines. This elevation can disrupt critical cellular pathways that regulate bone homeostasis, such as the Wnt signaling pathway [24]. Hyperglycemia can influence these pathways by modulating the activity of receptors, ligands, and antagonists, ultimately contributing to the development of osteoporosis [25]. Furthermore, these byproducts exhibit various activities, including the induction of cell death by reactive oxygen species and other substances, which further exacerbate the onset of osteoporosis. Moreover, when glucose concentrations increase, it triggers a rise in the release of inflammatory molecules. This disrupts the microenvironment that is crucial for normal bone metabolism. Concurrently, key substances that regulate bone formation, such as alkaline phosphatase (ALP), are inhibited under high glucose conditions, ultimately leading to the onset of osteoporosis [26]. In addition to diabetes, several other diseases can lead to secondary osteoporosis, such as Alzheimer’s disease [27]; however, the underlying mechanisms require further investigation. In addition to diseases, certain medications are also significant contributors to the onset of secondary osteoporosis [28]. Glucocorticoids are particularly representative of medications that induce osteoporosis [29]. When using glucocorticoids, it is essential to pay attention to the dosage and timing; otherwise, it is easy to cause osteoporosis. The mechanisms by which glucocorticoids induce osteoporosis are diverse, including reglulation of intestinal microbiota, cellular pathways, and effects on autophagy etc [30,31]. Glucocorticoids could affect osteoblasts, osteoclasts, and osteocytes [32]. Osteoporosis is one of the most serious side effects of glucocorticoids, but there is no effective treatment method till now.

### 3.3. The Role of Osteoblasts and Osteoclasts in Bone

In bones, osteoblasts primarily mediate bone formation, while osteoclasts primarily facilitate bone resorption. In a healthy body, these cells achieve a delicate dynamic balance to maintain bone mass and promote bone health [33]. However, factors such as disease can disrupt this balance, causing damage to bone health.

**Figure 2:**
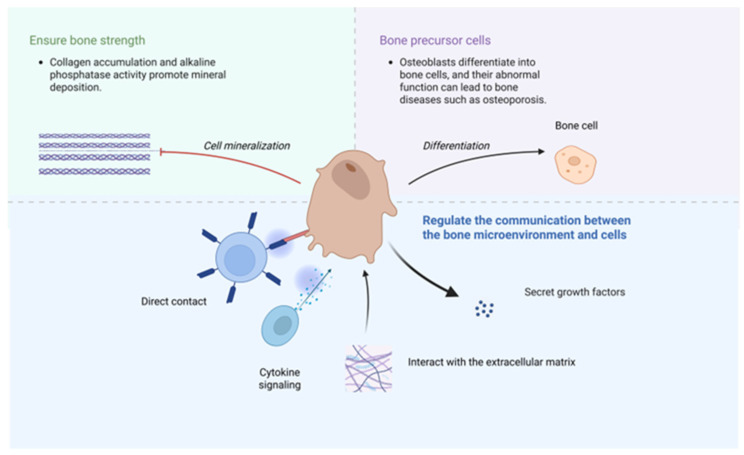
The role of osteoblasts in bone. Created in BioRender. lin, y. (2025) https://BioRender.com/dxqx3jl.

The influence of osteoblasts on bone health is multifaceted. First of all, the mineralization of osteoblasts is crucial for maintaining bone strength [34]. During osteoblast mineralization, collagen proteins accumulate and gradually form an orderly structural arrangement, while alkaline phosphatase facilitates mineral deposition. The degree of mineralization in the bone matrix is a key indicator of its quality, reflecting its intrinsic properties. Secondly, osteocytes are derived from osteoblasts, which are the most common cells in bones. Dysfunction of bone cells serves as the pathological foundation for several bone diseases, including osteoporosis. Osteoblasts can also establish close relationships with other cells, and facilitate intercellular communication through direct contact, cytokine signaling, and interactions with the extracellular matrix, and so on [35]. It can also secrete various growth factors, such as PDGF, BMPs, and IGFs, regulating bone health through multiple mechanisms [36] (Figure 2).

**Figure 3:**
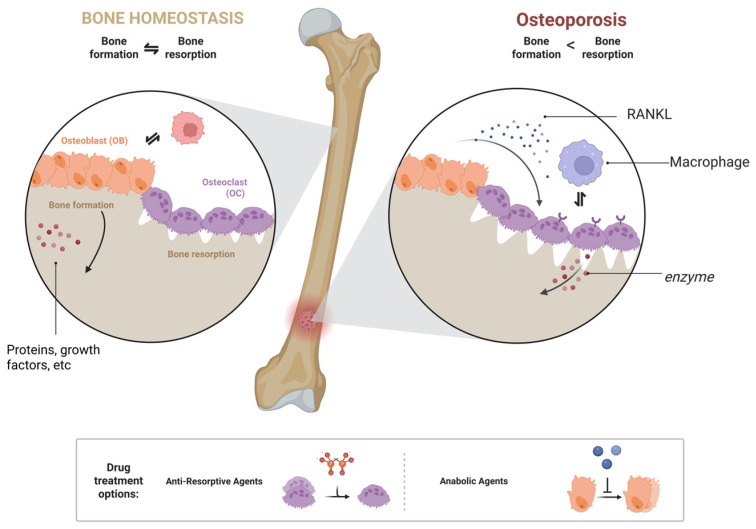
Briefly explain the pathogenesis of osteoporosis. Created in BioRender. lin, y. (2025), https://BioRender.com/v0namc6.

Similarly, osteoclasts can influence bones in various ways. Osteoclasts adhere to the surface of the bone matrix and secrete specific enzymes and proteic acids that degrade the bone matrix [37]. Additionally, its metabolism also plays a significant role in maintaining bone homeostasis. Metabolic disorders can lead to bone diseases such as osteoporosis [38]. Furthermore, the osteoclast-macrophage axis plays a significant role in bone immunity, and bone immune disorders can affect bone morphology [39]. Osteoblasts and osteoclasts jointly regulate bone health. Functional disorders or an imbalance between these cells are key contributors to osteoporosis (Figure 3).

### 3.4. Current Development Status of Existing Anti-Osteoporosis Drugs

The pathogenesis of osteoporosis mainly involves excessive bone resorption and inadequate bone formation, a multifaceted process that is challenging to fully address with conventional methods. Anti-osteoporosis drugs primarily target the two main pathogenic factors of osteoporosis: bone resorption and bone formation. Some drugs may exhibit dual activity. The clinical, widely applied anti-osteoporosis drugs are summarized in Table 2.

#### 3.4.1. Anti-Resorptive Agents

Anti-bone resorption drugs are medications that reduce bone mass loss by inhibiting the bone resorption activity of osteoclasts. They are the most widely used treatments for osteoporosis. Common anti-bone resorption drugs include bisphosphonates and receptor activator of nuclear factor kappa-B ligand (RANKL) inhibitors, etc.

Bisphosphonates were first discovered in the 1960s by Fleisch et al. Their biological activity has now developed to the third generation of bisphosphonates. In a meta-analysis of osteoporosis caused by glucocorticoids, researchers found that alendronic acid significantly increased bone density in the lumbar vertebrae, total hip, and other regions [41]. However, its effectiveness in reducing fractures may be related to factors such as race, and further investigation is still needed [52]. Bisphosphonates exhibit poor bioavailability and are rapidly excreted, necessitating high doses or prolonged administration. However, extending the dosing duration may increase side effects and significantly elevate the risk of fractures in patients. Consequently, the slow, controlled, and targeted release of bisphosphonates presents a significant challenge in their application.

Denosumab is a commonly used RANKL inhibitor. Compared to alendronate, denosumab reduces the risk of major osteoporotic fractures (MOF) by 39%. As the duration of treatment increases, the reduction in MOF risk gradually increases, from 9% in the first year to 31% in the fifth year [53]. However, it also has certain side effects. Taking Denosumab without subsequent treatment can lead to bone loss. Administering drugs such as alendronate after denosumab can help prevent this adverse effect [54]. This highlights the importance of combination therapy in reducing the toxicity and side effects of drugs.

#### 3.4.2. Anabolic Agents

Bone anabolic agents are drugs whose core function is to promote bone formation. Compared with anti-bone resorption agents, bone resorption anabolic agents are more suitable for patients with a significant decrease in bone density [55]. Parathyroid hormone drugs are commonly applied bone anabolic agents such as teriparatide and abalopapatide, etc.

Teriparatide is the 1–34 amino acid fragment of parathyroid hormone (PTH) and retains most of its biological activity. In a study involving postmenopausal patients with osteoporosis, 73 participants received 40 μg of teriparatide daily for a median treatment duration of 14 months. The results demonstrated that teriparatide treatment was associated with an average increase of 5.5% in lumbar bone mineral density. However, teriparatide is relatively high in cost of administration, requires subcutaneous administration, and is associated with side effects such as headache and joint pain; in rare cases, it may cause gynecomastia in men. Currently, in Europe, the maximum recommended duration of teriparatide treatment is 24 months [56]. This reduces drug dependence to some extent.

Abaloparatide is a peptide that selectively binds to the parathyroid hormone receptor. Its anabolic effect is more potent than that of teriparatide [57], making it a second-generation bone anabolic agent [58]. Clinical trial results show that, compared to 4.22% of patients in the placebo group who experienced vertebral fractures, the fracture rate in the abaloparatide group was only 0.58%, and the rate of non-vertebral fractures decreased from 4.7% in the placebo group to 2.7%. The increase in bone density was also significantly greater than that observed in the placebo group, demonstrating a strong anti-osteoporotic effect. Common adverse reactions include dizziness and headache. Compared with teriparatide, abaloparatide has fewer side effects and a lower incidence of hypercalcemia.

#### 3.4.3. Other Drugs

In addition to anti-bone resorption and bone anabolic agents, calcium supplements, vitamin D, and certain traditional Chinese medicine ingredients are also used in anti-osteoporosis treatments [59,60].

Calcium is an essential trace element vital for the human body, serving as a fundamental component of bone. Sufficient calcium intake is crucial for maintaining bone homeostasis. The absorption of calcium, along with other trace elements, is regulated by vitamin D, which enhances its bioavailability and directly influences bone metabolism to sustain bone health. These supplements are suitable for people lacking calcium, but they may also have certain side effects, including hypercalcemia, etc. In recent years, traditional Chinese medicine has increasingly played a significant role in the prevention and treatment of osteoporosis. Various traditional Chinese medicines and prescriptions have demonstrated effective results in managing osteoporosis [61,62]. In addition to pharmacological treatments, bone implants and lifestyle interventions are crucial for preventing and controlling osteoporosis. Maintaining a healthy lifestyle is also beneficial for preventing osteoporosis [63,64]. Additionally, innovative administration strategies may help mitigate the side effects of these drugs while enhancing their efficacy. For instance, transitioning from bisphosphonates to denosumab in postmenopausal women may alleviate the side effects associated with bisphosphonates [65], and combining estrogen with other anti-osteoporosis medications may further enhance their effectiveness in combating osteoporosis [66]. In addition, some drugs, such as romosozumab, have dual effects as both anti-bone resorption and anabolic agents [67,68]. Innovative treatment methods and drugs have demonstrated their advantages.

### 3.5. Bone Nanoparticles

Nanoparticles are an advanced drug-delivery system characterized by particle sizes at the nanoscale. These nanoparticles utilize specialized raw materials and techniques that enable targeted release at specific locations. Additionally, they protect the active ingredients from degradation and inactivation during transportation, storage, and in vivo conditions, thereby enhancing the stability of the drug. Additionally, nanoparticles can protect the active ingredients of the drug from degradation.

Nanocarrier preparations demonstrate significant potential in drug delivery and possess considerable application value in medicine [69] including bone-targeting drugs. From a structural perspective, nanoparticles can be broadly categorized into three components: carrier materials, ligand materials, and the loaded drugs. Additionally, some nanoparticles may incorporate excipients to further enhance their performance.

Bone-targeting nanoparticles can be classified into active and passive targeting based on their mechanisms [70]. Active targeting refers to achieving targeting by using targeting ligands, while passive targeting relies on the characteristics of the formulation itself, such as size and charge [71]. Based on the composition and preparation methods, the carrier materials, modified materials, and the synthesis methods are summarized below.

### 3.6. Carrier Materials

Carrier materials are nanoscale substances that transport drugs or bioactive molecules to specific regions of the bone. These carriers possess unique physical and chemical properties. With the ongoing advancements in nanotechnology, nanocarrier materials have emerged as a crucial component of bone-targeted drug delivery systems.

#### 3.6.1. Polymer

Polymers are a common type of nanoparticle materials in bone-targeted drug delivery systems, with polylactic-glycolic acid copolymer (PLGA) and chitosan serving as typical examples [72].

PLGA is a prominent polymer microsphere that holds a unique position in the medical field due to its exceptional biocompatibility and degradability [73,74]. It is widely utilized in surgical sutures, bone plates, and orthopedic implants. Its degradation products are relatively safe, and its degradation rate can be regulated. The physicochemical properties of PLGA are mainly related to its composition and molecular weight [75]. This ability allows it to inherit the beneficial properties of PLGA while also enabling the encapsulation of various drug molecules and providing sustained release properties. Consequently, PLGA is widely utilized in the medical field.

To overcome the poor targeting and high lipophilicity of the drug, researchers prepared a nano-formulation loaded with simvastatin using PLGA as the carrier. PLGA was used as the carrier material to prepare tetracycline-modified nanoparticles loaded with natural active compounds. PLGA not only provides a carrier framework for loading active compounds but also promotes the sustained release of drugs, thereby reducing their toxicity and side effects. Additionally, researchers precisely controlled its molecular weight and composition ratio, maintaining the nanoparticle size at approximately 200 nm, which enhances cellular uptake of the nanoparticles [76]; the adjustability of PLGA also plays a crucial role.

Chitosan is a natural polymer and [77]. Its structure contains a number of amino and hydroxyl groups, which can combine with common bone-targeted agents, thereby endowing them with bone-targeted properties [78]. Due to its natural biocompatibility, biodegradability, and low immunogenicity, chitosan holds great potential for use in bone transplantation, offering a novel solution to the challenges posed by bone diseases [79].

Chen et al. prepared a chitosan-based nanoparticle with an obvious therapeutic effect on osteoporosis in OVX mouse models [80]. Additionally, researchers designed chitosan nanomaterials loaded with growth factors and incorporated them into fibrous scaffolds. The results showed that it could effectively promote cartilage regeneration [81]. Similarly, in another study, researchers incorporated BMP2 plasmid DNA into chitosan nanoparticles, which were then embedded into hydrogels that had the ability to accelerate the healing of bone defects [82]. Chitosan nanomaterials are also widely used for delivering hormone-like substances. Researchers have utilized chitosan to deliver melatonin and modified it with the conductive material PAP, demonstrating excellent biological properties [83]. As a natural substance, chitosan has significant advantages in terms of safety and biocompatibility.

#### 3.6.2. Liposomes

Liposomes are vesicular structures composed of phospholipid bilayers. They are the most widely used drug carriers in nanoparticle-mediated delivery systems. These nanoparticles consist of cholesterol, phospholipids, PEG lipids, and, most importantly, ionizable cationic lipids. Lipid nanoparticles have demonstrated effectiveness and versatility in delivering siRNA and various other RNA molecules [84]. The first FDA-approved RNA interference therapy was also based on the implementation of nanoparticles [85]. As a carrier, liposomes offer numerous advantages, including a cell-like membrane structure, high biocompatibility, low immunogenicity, protection of drugs or active compounds, extended drug half-life, reduced toxicity, and enhanced efficacy [86].

In a recent study, a liposome-based nanomaterial (CH6-LNPs-siNLRP3) for the treatment of osteoporosis was prepared. The study indicates that liposome-based CH6-LNPs-siNLRP3 do not cause cytotoxicity even at higher concentrations due to their high biocompatibility. The experimental results show that parameters such as bone mineral density (BMD), bone volume fraction (BV/TV), and trabecular bone count (Tb.N) in rats showed significant improvement [87]. The nanoparticles exhibit excellent siRNA loading capacity, ensuring stable delivery and effective functional performance of siRNA when used with liposomes as the carrier material.

#### 3.6.3. Metal Nanoparticles

MOF is a cutting-edge nanomedicine delivery platform, which has attracted widespread attention from the global medical and scientific communities [88]. Their unique structures enable the precise delivery of drug molecules, facilitating effective disease treatment. Additionally, lanthanide metal nanoparticles have emerged as preferred materials for tissue engineering due to their distinctive properties [89].

Although earlier studies indicated that metal nanoparticles could pose a risk of toxicity in earlier studies, further research has demonstrated that they can be safe and beneficial when used at appropriate sizes and dosages. Precious metal nanoparticles have made significant advancements in recent years, demonstrating considerable potential. Gold nanoparticles (AuNPs) are outstanding representatives of precious-metal nanoparticles, which have been utilized in biology and clinical medicine for many years and have been developed to regulate both osteogenic differentiations. With the assistance of gold nanoformulations, miRNA can more effectively enter the cytoplasm to exert its functions [90]. Gold nanoparticles also help regulate the intestinal flora, thereby indirectly influencing osteoporosis [91]. In another study, researchers modified gold nanoparticles with bisphosphonates, which significantly inhibited osteoclast differentiation [92]. Silver nanoparticles also exhibit impressive performance and are primarily employed for antibacterial, anticancer, bone induction, bone regeneration, and other applications [93]. They can regulate the proliferation and differentiation of mesenchymal stem cells (MSCs) involved in bone regeneration. Additionally, magnetic nanoparticles possess unique physical properties and can be combined with static magnetic fields, yielding positive results in promoting wound healing and bone regeneration.

#### 3.6.4. Hydroxyapatite

Hydroxyapatite is a crucial component of bones and exhibits excellent biocompatibility [94]. It is extensively utilized in the field of bone implants. Through surface modification techniques, the surface of hydroxyapatite nanoparticles can be tailored to fulfill various biological functions [95].

These nanoparticles serve as initiators for bone formation. However, hydroxyapatite has certain limitations in its applications, including poor mechanical properties, susceptibility to damage under significant external forces, and a lack of antibacterial properties, which increases the risk of infection. To address these issues, researchers have employed cation doping in hydroxyapatite to mitigate these deficiencies and enhance its application value [96]. Studies have indicated that excessive local doses of hydroxyapatite nanoparticles can disrupt the microenvironment, while systemic administration has minimal effects [97]; therefore, it is crucial to consider potential side effects when using them.

Researchers aim to overcome the limitations of peptide drugs, such as poor oral bioavailability, by using hydroxyapatite. Darsheen J. Kotak et al. prepared hydroxyapatite nanoparticles loaded with salmon calcitonin (SCT) [98]. Compared to subcutaneous injection of free SCT, the mucosal permeability and targeting of sublingual administration of SCT-HAP-NPs significantly increased. Similarly, hydroxyapatite nanoparticles loaded with daidzein were designed, and the experimental results demonstrated their excellent therapeutic effects on postmenopausal osteoporosis [99]. Unlike other carrier materials, hydroxyapatite itself has certain bone-targeting properties. Therefore, some hydroxyapatite nanoparticles do not require additional bone-targeting agents, and their synthesis methods are relatively simple.

#### 3.6.5. Other Carrier Materials

Carrier materials play a crucial role in nanoparticles. In addition to the various materials highlighted in this paper, nanoparticles can also utilize other carrier materials, such as mesoporous silica, and polyelectrolyte [100,101], and some nanoparticle carriers use a variety of materials, encompassing both organic and inorganic sources [102]. These materials collectively contribute to the support of different types of nanoparticles.

### 3.7. Modified Materials

The modified materials used in nano preparations exhibit specific biocompatibility, demonstrate affinity for the target site, and not compromise the efficacy of the drug. Common ligand modification materials can be categorized into bisphosphonates, tetracyclines, and other compounds. Ligand materials can be understood as bone-seeking agents, enabling nano preparations modified with them to be accurately delivered to bone tissue.

#### 3.7.1. Bisphosphonates

As a widely used bone-targeted ligand, bisphosphonates have strong binding affinity toward the primary component of bone. From the perspective of molecular structure, bisphosphonates consist of two phosphate groups and a hydroxyl side chain, which bind to hydroxyapatite through bidentate or tridentate chelation [103]. Research indicates that the side chain significantly influences the affinity between bisphosphonates and bone. Further studies have demonstrated that bisphosphonates containing one hydroxyl group and two phosphate groups can bind to all types of hydroxyapatite.

In recent years, bone-targeting nanoparticles modified with alendronic acid have garnered significant attention. Researchers have covalently bound alendronic acid to liposomes, and the results show that alendronic acid can increase the concentration of nanoparticles at the bone site several times, and it can prolong the bone retention time of the drug. Rizzi et al. modified nanomicelles with alendronic acid, which exhibited strong targeting capabilities [104]. Similarly, Sun et al. prepared an alendronic acid-functionalized polymer micelle, which effectively delivered the flavonoid icaritin to the target site [105]. The results of these investigations demonstrate that alendronate significantly increases the affinity of nanoparticles for hydroxyapatite, thereby enhancing drug delivery efficiency and bioavailability.

#### 3.7.2. Tetracycline

Tetracyclines are characterized by a common chemical structure consisting of four (tetra-)linear fused hydrocarbon rings. The tetracycline nucleus can be modified by attaching various functional groups, which influence its properties [106]. Additionally, research indicates that tetracyclines may have a lasting impact on bone metabolism.

In the study of the mechanism of action between tetracycline and bone, researchers initially believed that tetracycline could directly interact with and bind to hydroxyapatite, the primary component of bone [107]. Later studies revealed that the binding mechanism of alizarin to bone involves chelation with positively charged bivalent or trivalent ions. Although the structure of tetracycline is more complex than that of alizarin, its binding mechanism with bone is thought to be similar. However, this perspective has not been universally accepted within the academic community, and some researchers argue that the interaction is primarily due to tetracycline’s binding with proteins, such as collagen, among others.

The ability of tetracycline to bind effectively to bone has been thoroughly validated in clinical studies. Some pregnant women who take tetracycline may give birth to infants with yellow teeth, a condition resulting from the interaction between tetracycline and developing teeth. Tetracycline-labeled bone biopsy is the gold standard for diagnosing renal osteodystrophy [108]. In recent years, tetracycline-modified bone-targeted nanoparticles have been extensively investigated.

Researchers modified PLGA nanoparticles with tetracycline. Experimental results demonstrated that, compared to unmodified nanoparticles, TC-PLGA NPs significantly enhanced targeting and therapeutic efficacy without affecting their fundamental physicochemical properties, specifically, compared to nanoparticles without tetracycline conjugation, tetracycline enhanced the aggregation of nanoparticles at the bone site [76]. It demonstrates the efficient binding of tetracycline to bones.

#### 3.7.3. Amino Acids and Oligopeptides

Amino acids and oligopeptides are effective bone-targeting agents, among which aspartic acid is a relatively representative one. The surface of it contains functional groups and is prone to a negative charge. Bones contain a relatively large number of calcium ions, which can have an affinity with aspartic acid through charge interaction. This affinity may be related to the number of residues. The more residual samples are exposed, the greater the affinity with the bone [109]. Moreover, these functional groups, such as amino and carboxyl groups, can be modified relatively easily through chemical coupling and other methods and readily bind to bone-targeting carriers [110]. The derivatives of these amino acids also have strong affinity for bones. For instance, a bone-targeting peptide (AspSerSer, DSS)_6_ can be loaded onto carrier materials through techniques such as click chemistry, thus achieving precise targeting [111].

Bone proteins are also commonly used as bone-targeting agents. These proteins have special amino acid sequences, such as γ-carboxyl glutamic acid residues [112]. These special structures have a strong affinity for components such as hydroxyapatite and collagen in bones, enabling the formulation to precisely anchor in bone tissue and thereby enhancing the targeting ability. Amino acids and polypeptides, as natural substances, have high biological affinity and enzymatic hydrolysis properties, and they do not cause toxicity.

Researchers modified PEG-PLGA with (Aspartic acid)_3_, which can effectively target bone tissue, enhance the drug’s bioavailability, and enable targeted release [113]. Similarly, Xue Liu et al. modified the nanoformulator with the Arg-Gly-Asp peptide and developed a targeted delivery system that enhances uptake by osteoblasts [114]. In addition, these experimental results indicate that the toxicity of these nanoparticles is minimal under specific conditions. Amino acids and polypeptides, as natural substances, have high biological affinity and enzymatic hydrolysis properties, and they do not cause toxicity.

#### 3.7.4. Surface Modifications

The surface properties of nanoparticles are critical parameters that influence their stability, safety and biological activity. For instance, nanoparticles with varying structures may exhibit different levels of toxicity, and the body’s absorption of these particles is influenced by their size. In addition, surface properties can also influence the protein corona [115]. The protein corona significantly affects the efficacy of nanomedicines and may cause a series of adverse effects, such as reduced targeting. It is important to note that while protein adsorption can lead to complications, this characteristic can also be leveraged to modify nanoparticles with active proteins, thereby reducing toxicity and enhancing their duration of action and targeting capabilities. Generally speaking, nanomedicines with a negative surface charge have poor biological activity owing to their difficulty in adhering to anionic cell surfaces. Therefore, surface modification is often required. The surface charge properties of nanomedicines can be changed by introducing other positively charged materials, thereby improving their targeting and efficacy. For instance, PLGA nanoparticles typically possess a negative charge on their surface, which can be altered to a positive charge through the use of cationic polymers. These positively charged PLGA nanoparticles enhance interactions with cell membranes, thereby increasing their effectiveness [116]. Therefore, by appropriately modifying the surface to alter the size, roughness, charge, and other properties of nanoparticles, their therapeutic effects can be enhanced. This necessitates in-depth studies by researchers on the properties of raw materials and the mechanisms of their interactions with cells.

#### 3.7.5. Other Finishing Materials

In addition to the aforementioned modification materials, nanoparticles can also have a variety of other modification materials, such as peptides and growth factors. Although these modification materials differ in their chemical structure, they all exhibit certain levels of biocompatibility and targeting capability.

### 3.8. Synthesis Methods of Nanoparticles

The preparation of bone-targeting nanomedicines generally involves two parts: one is the preparation of conventional nanoparticles, and the other is the connection of targeting groups. The targeted group connection method can be classified into two types. Generally, one is to prepare nanoparticles first and then modify them with targeted groups, and the other is to modify the raw materials with targeted groups before preparing the nanoparticles. Although the sequence differs, the underlying mechanism is similar.

#### 3.8.1. Emulsion Solvent Evaporation Method

The emulsified solvent volatilization method is a widely used technique for preparing bone-targeting nanoparticles, particularly in laboratory settings. This method involves dissolving raw materials in two immiscible solvents, typically an aqueous phase and an organic phase, and then making an emulsion by mechanical agitation or ultrasonic emulsification, followed by the removal of the organic phase to yield nanoparticles. While single emulsions often exhibit low drug encapsulation rates, employing a double emulsion can enhance embedding efficiency [117,118]. This technique boasts a high encapsulation rate, good reproducibility, and does not necessitate complex instrumentation.

#### 3.8.2. Self-Assembly Method

The self-assembly method is a technique that spontaneously forms ordered structures from basic structural units without external forced intervention. Instead, it arranges them into ordered aggregates based on hydrogen bonds, van der Waals forces, electrostatic effects, etc. Its operation is relatively simple and does not require excessive external intervention [119]. However, it has many design forces, which may lead to significant deviations.

#### 3.8.3. Green Synthesis Method

Green synthesis methods offer distinct advantages, utilizing various biological agents, such as plant, bacterial, and fungal extracts, due to their biocompatibility. Plant extracts, in particular, hold significant value in the green synthesis of nanoparticles for several reasons. Firstly, they are more cost-effective and do not require complex methods, such as separation, culturing, and maintenance, making them relatively easy to source and prepare. Secondly, plant-based formulations are time-efficient and facilitate the large-scale production of nanoparticles, thereby significantly reducing time-related costs. Additionally, plant extracts typically contain certain reducing components, allowing for the green synthesis of nanoparticles through redox reactions [120].

#### 3.8.4. Other Synthesis Methods

There are numerous techniques for preparing nanometer-scale materials, which will not be described in detail here. Different methods have different suitable environments and address the needs under different conditions.

### 3.9. Targeted Group Connection Method

#### 3.9.1. Covalent Binding Method

Covalent binding is a widely used technique for linking targeted ligands. The target groups are linked by forming stable covalent bonds between the surface of the nanocarrier or nanoparticles and the target groups through chemical reactions. For instance, the alendronic acid salt substructure contains amino groups, phosphonic acid groups, which can naturally react with carboxylic acid groups, hydroxyl groups, thereby achieving coupling. Zheng et al. prepared iron oxide nanoparticles by the post-modification method and coated them with polyglucose-sorbitol-carboxymethyl ether (PSC). The PSC contained certain carboxylic acid groups. After activation of the carboxylic acid groups, the two could combine well. The characterization results showed that approximately 27 μg of alendronate could be coupled with 1 mg of nanoparticles, and the binding of alendronate did not affect the crystallinity or morphology of the nanoparticles [121]. This method precisely controls the binding ratio of the target group to the carrier, which, to a certain extent, increases yield, reduces preparation costs, and ensures a stable connection that is not easily damaged.

#### 3.9.2. Electrostatic Adsorption Method

Electrostatic adsorption is also a common method that relies solely on the interaction forces between charges to maintain the combined state. Weng et al. first synthesized CaF_2_@Mg-MOF (CM) nanoparticles and then functionalized the CM nanoparticles with amino groups. In an appropriate aqueous environment, the amino groups can be protonated into -NH_3_^+^, thereby endowing the entire nanoparticle surface with a positive charge. In addition, sodium alendronate (Ald) was anchored to polyacrylic acid (PAA), a polyelectrolyte containing carboxyl groups. These carboxyl groups both react with sodium alendronate to anchor it to PAA and hydrolyze to generate a negative charge. Enable PAA-Ald to spontaneously and firmly coat the surface of CM-NH_2_ through electrostatic adsorption, forming bone-targeted nanoparticles [122]. The physical adsorption method is simple to operate and retains the biological activity of the targeted carrier and nanoparticles. However, because it relies on non-covalent interactions, the binding force is relatively weak and can be easily affected by pH value, ionic strength, etc.

### 3.10. Bone-Targeting Nanoparticles: Acting Mechanisms on Osteoporosis

#### 3.10.1. Bone-Targeting Nanoparticles for Inhibiting Oxidative Stress in Osteoblasts

Bone-targeting nanoparticles can mitigate oxidative stress through several mechanisms. Oxidative stress is one of the significant factors that lead to osteoporosis. Oxidative stress not only contributes to osteoporosis but also causes other bone diseases. It can induce apoptosis, such as ferroptosis, DNA damage, and mitochondrial damage, thereby negatively impacting bone health [123]. They are by-products of cellular respiration and are primarily produced by mitochondria. Additionally, endoplasmic reticulum stress and other factors can cause the production of ROS.

Inflammation is closely linked to oxidative stress, and the two interact and influence one another. Inflammatory responses can contribute to the occurrence of osteoporosis. Inflammation is regulated by macrophages. Different macrophage subtypes have distinct effects on inflammation. Certain nanoparticles can decrease M1 polarization of macrophages, thereby reducing oxidative stress [124,125,126].

The activity of antioxidant enzymes can be regulated by certain nanomaterials, which enhance their function and inhibit oxidative stress. Research has demonstrated that certain nano-coatings can shield osteoblasts from oxidative stress by either mimicking the function of antioxidant enzymes or enhancing their activity in vivo [127]. However, their effects in preventing osteoporosis still need further investigation.

High levels of inflammatory factors can inhibit the differentiation of osteoblasts. Bone-targeting preparations can reduce the levels of NLRP3 inflammasome and pro-inflammatory cytokines IL-1β and IL-18 in osteoblasts. By lowering the levels of these pro-inflammatory factors, effectively reducing the inhibitory effect of inflammation on osteoblasts [87]. In recent years, the treatment of osteoporosis through the bone-gut axis has shown great potential. Intestinal diseases can damage the body’s absorption of calcium and other substances, trigger inflammatory responses, and thereby harm bone health. Nanomedicines can treat osteoporosis by regulating the intestine. The integrity of the intestinal barrier has a significant impact on its homeostasis, which depends on proteins such as claudin-1 and ZO-1. Nanoformulations can significantly increase the expression levels of these proteins in the intestine. In addition, nanomaterials can influence the intestinal tract by maintaining intestinal health through the abundance of specific microbial populations. By improving the systemic inflammatory environment, ultimately alleviating osteoporosis [91,128].

Some nanoparticles are infused with natural products that exhibit strong antioxidant properties. Some natural chemical compounds containing phenolic hydroxyl, sulfhydryl, and other functional groups can directly contribute to the removal of free radicals [129]. Nanotechnology offers a promising drug-delivery system capable of encapsulating these compounds to mitigate oxidative stress in osteoblasts. In addition to delivering these drugs or natural products, nanoformulations can protect them from inactivation due to degradation and other conditions. Research indicates that the efficacy of nanoparticles loaded with natural chemical compounds is significantly superior to that of using the compounds alone [130]. This highlights the potential applications of nanoparticles in drug delivery systems.

#### 3.10.2. Nanoparticles That Promote Osteoblast Proliferation and Differentiation

The proliferation and differentiation of osteoblasts are influenced by a variety of factors. The proliferation and differentiation of osteoblasts profoundly impact bone homeostasis. The mineralization of osteoblasts is a critical process for bone formation. And this process can control various physiological processes and make bones a compact structure. Mineralized nodules are the primary manifestation of osteoblasts performing osteogenic functions and serve as an important indicator of their differentiation and maturation.

Bone-targeting nanoparticles can promote the proliferation and differentiation of osteoblasts by modulating signaling pathways. A variety of proteins play crucial roles in the proliferation and differentiation of osteoblasts, and the expression of these proteins is regulated by certain cellular pathways, ultimately affecting osteoblast proliferation and differentiation. For instance, sclerostin is a potential target for enhancing bone formation in conditions such as osteoporosis and during the bone healing process. It is regulated by the Wnt signaling pathway, a crucial regulator of bone formation [131]. In addition to sclerostin, proteins such as PINK1 and BMP2 are also influenced by these signaling pathways and play a role in osteoblast proliferation and differentiation [132,133]. Furthermore, certain signaling pathways can contribute to various diseases; for example, dysregulation of the Wnt/β-catenin signaling pathway can lead not only to bone diseases, but also to neurodegenerative diseases and hair loss. Other factors, such as hyperglycemia, can disrupt these signaling pathways, resulting in secondary osteoporosis. The properties of nanoparticles offer a novel therapeutic approach for managing secondary osteoporosis.

Oxidative stress also significantly impacts the proliferation and differentiation of osteoblasts. It inhibits these processes by down-regulating the Wnt/β-catenin signaling pathway and β-catenin expression, as well as by inhibiting the FOXO transcription factor [134]. Nanoparticles can mitigate oxidative stress in the previously described way, thereby promoting osteoblast proliferation and differentiation [135,136,137].

In addition, certain nanoparticles can regulate autophagy in cells, and this effect is influenced by the morphology of nanoparticles. They affect the expression of specific cellular pathways by regulating autophagy in cells, thereby promoting the differentiation of osteoblasts. Additionally, as a self-protective process, autophagy helps eliminate intracellular reactive oxygen species and other harmful substances, further facilitating the proliferation and differentiation of osteoblasts [138]. Wang et al. prepared hydroxyapatite nanoparticles and demonstrated that nanoparticles increased the LC3II/LC3I ratio and stimulated autophagy through the mTOR signaling pathway, and this process promoted the differentiation of osteoblasts [138]. Bone-targeting nanoparticles can enhance osteoblast differentiation by modulating autophagy through specific signaling pathways, offering a novel approach for the treatment of osteoporosis.

#### 3.10.3. Anti-Resorptive Therapy

Osteoclasts originate from bone marrow-derived macrophages (BMMs), and their proliferation and differentiation are influenced by signaling pathways such as mitogen-activated protein kinase (MAPK) and nuclear factor κB (NF-κB). Nanoparticles can inhibit the phosphorylation of these cellular pathways, thereby blocking downstream signaling [139]. Reduce the activation of core transcription factors for osteoclast differentiation, such as c-Fos, and prevent the expression of downstream target genes. Meanwhile, cytokines such as RANKL, NFATc1, and M-CSF are also among the factors inducing osteoclast differentiation [140]. Besides reducing its expression, nanoparticles can prevent the differentiation of osteoclasts by blocking the interaction between receptor activators and receptors. Autophagy is an important pathway to inhibit the differentiation of osteoclasts. Nanomaterials can enhance the expression of genes such as p62 and LC3β and inhibit the proliferation and differentiation of osteoclasts by promoting autophagy [141].

Apoptosis is also induced by cytokines. Nanoparticles can increase the expression levels of pro-apoptotic proteins such as Bax and Cleaved-caspase 3, inhibit the expression levels of anti-apoptotic proteins such as Bcl-2, and promote osteoclast apoptosis. This inhibits bone resorption by osteoclasts. It is not only induced by cytokines but also strongly associated with the level of oxidative stress [142]. An appropriate level of ROS induces osteoblast senescence and excessive production of RANKL, which promotes the generation of more osteoclasts [143,144]. In addition, excessive ROS induces inflammatory responses, which are conducive to osteoclast differentiation. However, excessive accumulation or reduction in ROS can inhibit its survival. On one hand, nanomaterials can play a role similar to that of oxidase, increasing the ROS level of osteoclasts, inducing oxidative stress, and accelerating osteoclast apoptosis [145]. On the other hand, nanomedicines can also inhibit the proliferation and differentiation of osteoclasts by inhibiting the production of ROS. The survival of osteoclasts depends on an acidic microenvironment, and the substances they secrete, such as protons, further intensify the acidification of the system, creating a vicious cycle in patients with osteoporosis, exacerbating bone resorption. Based on this characteristic, the pH-responsive release of nano-preparations can be achieved. Meanwhile, some alkaline nanomaterials, such as calcium carbonate nanoparticles and sodium bicarbonate nanoparticles, can neutralize the acidic environment in the body, promote osteoclast apoptosis, and inhibit bone loss [146,147].

Apart from acidic conditions, other structures, such as the F-actin ring, are crucial for osteoclasts to perform bone resorption functions. The F-actin ring creates a sealed environment on the bone surface and serves as a critical site where osteoclasts secrete substances, such as protonic acid, to carry out their functions. The structure and dynamic changes in the actin ring may vary under different bone resorption modes, thereby regulating the movement and bone resorption efficiency of osteoclasts. The actin ring is a key structure for bone resorption in osteoclasts. Nanoparticles can affect the function of osteoclasts by inhibiting the expression of structures such as the actin ring [148]. 

#### 3.10.4. Nanoparticles Based on RNA Interference

RNA interference (RNAi) is an emerging therapeutic approach that was first proposed by Mello and Fire [149]. However, research in the field has experienced fluctuations, and it was not until 2018 that the first RNAi therapy received approval from the FDA. The overexpression of certain genes can contribute to osteoporosis. The application of bone-targeting nanoparticles can effectively deliver siRNA, enabling it to accumulate near target cells and silence associated genes, thereby improving the condition of osteoporosis [150].

MiRNA plays a crucial role in regulating gene expression. As a short non-coding RNA molecule composed of a limited number of nucleotides, miRNA can bind to complementary mRNA, inhibiting ribosomes from translating its sequence and thereby silencing the mRNA. Additionally, it can directly promote mRNA degradation, leading to alterations in gene expression and consequently regulating various physiological processes within the body. By finely regulating gene expression, miRNA ultimately influences cellular activities [151,152]. MiRNA plays an important role in regulating gene expression. Researchers estimate that approximately 60% of human protein-coding genes may be regulated by miRNA. Consequently, the targeted delivery of miRNA to specific cells to modulate gene expression holds significant potential for the treatment of osteoporosis. It can impact osteoblasts by enhancing osteogenic differentiation and inhibiting inflammation [153]. However, the short sequence of miRNA and the lack of specific ligands can result in poor targeting. siRNA is a short-chain RNA molecule. It is a key molecule in the RNA interference pathway, binding to specific mRNA and guiding RNA-induced silencing complexes to recognize and cleave target mRNA, thereby inhibiting their translation and achieving gene silencing. Short interfering RNA inhibits gene expression through a highly regulated enzyme-mediated process known as RNA interference (RNAi). The specific delivery of microRNA through bone-targeted nanomaterials presents substantial clinical value. Recent investigation has demonstrated that specific aptamers can enhance the targeted uptake of certain siRNAs by osteoblasts in vitro through the process of endocytosis, thereby facilitating precise gene silencing in these cells.

The nanoparticle can deliver miRNA and siRNA to osteoblasts and other cells in a targeted manner, thereby exerting various functions, such as regulating gene expression and osteoblast activity, while overcoming the limitations associated with the targeting and side effects of miRNA and siRNA. Zhang et al. prepared a polyelectrolyte nanoparticle capable of effectively interfering with the expression of Dcstamp mRNA. The results of animal experiments demonstrated that this nanoparticle significantly reversed osteoporosis symptoms in mice [101]. It precisely achieved the specific silencing of mRNA, providing technical support for RNAi therapy in osteoporosis.

RNAi therapy possesses several advantageous characteristics. Firstly, it is highly effective; once a gene is successfully silenced, it can often continue to exert its influence. Additionally, RNAi therapy offers precise targeting, minimizing interference with the expression of normal genes and thereby reducing toxic side effects. It is believed that with ongoing technological advancements, RNAi-based nanocarriers will play an increasingly significant role in the management of osteoporosis.

#### 3.10.5. Bone-Targeting Nanoparticles for Supplying Trace Elements

Certain trace elements are essential for maintaining bone homeostasis. In addition to serving as raw materials for bones, trace elements can also regulate signaling pathways such as NF-κB and PI3K-Akt, inhibit the release of pro-inflammatory factors, and promote the differentiation of osteoblasts. It has functions such as inhibiting the activity of osteoclasts [154,155,156]. Researchers have integrated trace elements, such as zinc and magnesium, into nanomaterials, which not only preserve the excellent properties of nanomaterials but also deliver them to bone tissue to better release those and boost their absorption by the body, bringing new breakthroughs in the treatment of diseases such as osteoporosis. The use of nanoparticles facilitates the slow release of trace elements, thereby increasing patient compliance. Based on the acidic bone microenvironment, a series of nano-preparations containing calcium and magnesium ions can be designed, such as calcium carbonate nano-preparations and magnesium organic frameworks coated with CaF_2_ (Mg-MOF), achieving the purpose of precisely supplementing trace elements to treat osteoporosis. To improve the bone microenvironment, it provides a material basis for promoting bone formation and other aspects [122]. The nanoparticles with trace elements fully exploit the synergistic advantages of the carrier and its components and aligns with the pathological basis of osteoporosis.

#### 3.10.6. Enhanced Osteoblast Adhesion

Cell adhesion is a process by which cells attach to other cells or the extracellular matrix, mediated by cell surface receptor molecules [157]. Additionally, certain inorganic ions can influence cell adhesion [158]. The strength of cell adhesion during cell culture is crucial for assessing the health status of cells. When conventional drugs and treatment methods fail to cure a disease, orthopedic implants are often used as an alternative treatment approach. However, this may lead to rejection, and the lifetime of orthopedic implants is relatively short, increasing the economic burden on patients. In the treatment of bone-related diseases, the level of cell adhesion significantly impacts the effectiveness of implants and reflects their biocompatibility. Moreover, the normal level of osteoblast adhesion is vital for the process of bone repair.

The surface roughness of different preparations influences osteoblast adhesion in varying ways. In particular, nanoscale roughness closely resembles the nanostructure of the natural extracellular matrix, which promotes osteoblast adhesion. Specifically, a sample with a root-mean-square roughness of 30 nm closely approximates the roughness of bone surfaces and exhibits the highest number of adhering cells, making it more conducive to osteoblast adhesion than nanoparticles with larger surface areas. Additionally, the surface chemical composition of nanoparticles is another potential mechanism influencing osteoblast adhesion; however, the specific mechanism remains to be further researched.

It is important to note that normal cell adhesion exerts a critical function in bone growth and the stability of bone homeostasis. However, excessive adhesion can lead to adverse effects, such as the promotion of factors that induce osteoclast differentiation. Therefore, it is essential to explore methods to accurately regulate osteoblast adhesion, enhancing attachment while mitigating its negative consequences.

#### 3.10.7. Other Mechanisms

**Figure 4:**
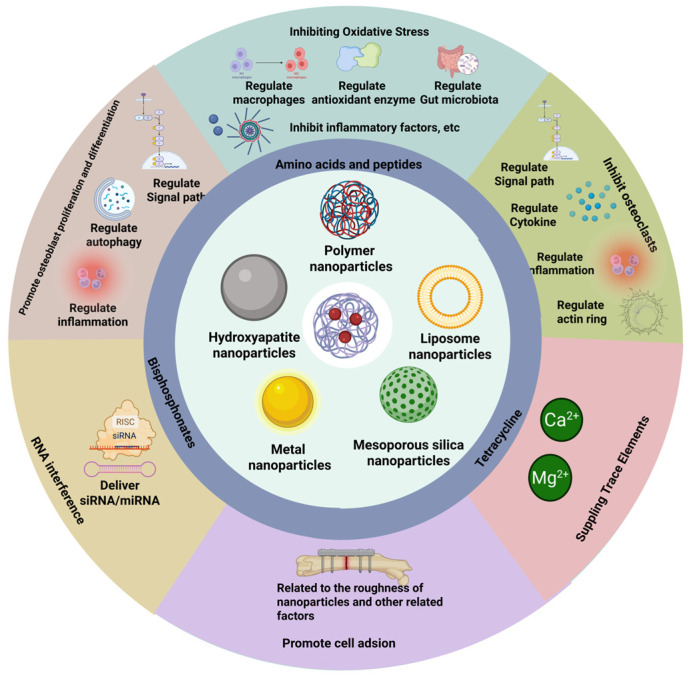
Bone-targeting nanoparticles acting on osteoblasts. Created in BioRender. lin, y. (2025), https://BioRender.com/s0nqlx3.

In addition to the mechanisms of action of bone-targeting nanoparticles that has been emphasized above, nanoparticles can also prevent osteoporosis or the side effects of drugs through multiple mechanisms such as regulating the mitochondrial respiratory chain, inhibiting endoplasmic reticulum stress, regulating the microenvironment of osteoporosis, and promoting osteoprotegerin (OPG), CSF2, CCL2, etc. Many nanoparticles also have multiple mechanisms of action simultaneously. They jointly play a role in treating osteoporosis. Bone-targeting nanoparticles achieve specific binding to bones through targeted ligands and deliver drugs via carrier materials to produce various therapeutic effects, thereby effectively treating osteoporosis (Figure 4). The information on raw materials, targeting methods, and mechanisms of action of bone-targeting nanoparticles is presented in Table 3.

### 3.11. Clinical Application Predicaments

Although bone-targeting nanoparticles have demonstrated significant potential for treating osteoporosis, the vast majority of them remain in the preclinical research stage, and their clinical translation faces numerous challenges.

#### 3.11.1. Safety Issues

In drug delivery systems, safety is an important requirement for bone-targeted nanoparticles. Ideally, these nanoparticles should only accumulate at the target site, and both the nanoparticles and their degradation products should be non-toxic, eventually being excreted from the body through metabolism. However, there are still considerable challenges to the safety of nanoparticles at present.

As artificial substances, nanoparticles can activate the immune system and trigger rejection responses in the body [178]. In addition, some nanoparticles exhibit direct cytotoxicity. For instance, researchers have found that silver nanoparticles can activate the iNOS-NO-RNS signaling pathway, induce the generation of NO, and cause the death of osteoblasts [179]. The safety of bone-targeted nanoparticles after degradation also requires further verification. For example, PLGA is a common material for bone-targeted nanoparticles. Its degradation products are lactic acid and glycolic acid. These two substances are relatively safe, but if the dosage is inappropriate, they may still cause a series of adverse effects, such as inflammation and osteoblast death. This is not in contradiction with the original therapeutic intention of bone-targeted nanoparticles. By further studying its mechanism of action, optimizing raw materials, and controlling dosage, its toxicity can be minimized to the greatest extent. Furthermore, existing evidence indicates that bone-targeted nanoparticles accumulate not only at the bone site but also in secondary organs such as the liver and heart.

In addition, many current experimental designs have certain safety issues. Drugs need to reach the target site through the plasma, and bones are highly vascularized organs. The composition of plasma varies greatly among different species. The degree of cellular uptake and aggregation of the same nanoparticle in different animal models also differs, which leads to different human applicability of preclinical data [180]. If clinical trials are directly conducted based on animal experiment data, adverse reactions that have not been observed in animal experiments may occur in humans, threatening the health of the subjects. If clinical trials are conducted solely on the basis of animal experiment data, adverse reactions not observed in animal experiments may occur in humans, potentially endangering the health of the subjects.

#### 3.11.2. Regulatory Issues

There is no unified definition of nanomaterials worldwide, nor are there authoritative and consistent regulatory guidelines for nanomedicines. There are significant differences and even dynamic adjustments in regulatory standards among different countries and individual cases. The same nanomedicine may be classified as a drug or medical device in different regions. This lack of uniformity in standards significantly increases the complexity and uncertainty of the approval and regulation of nanomedicines, and some guidelines or standards lack legal effect. In addition, the production process for nanoparticles is complex, making it difficult to ensure the stability and consistency of product quality. Their unique physical and chemical properties, biological interaction characteristics, and potential toxicity impose higher demands on quality control and further complicate regulatory approval. Although the International Council for Harmonization of Technical Requirements for Pharmaceuticals for Human Use (ICH) has sought to promote international coordination in the regulation of nanomedicines, a unified reporting standard has not yet been established [181]. This not only delays the application cycle for new clinical trials of nanomedicines by several months compared to that for traditional drugs but also further undermines patients’ trust in nanomedicines.

#### 3.11.3. Cost Issues

The process from drug research and development investment to clinical application is long and complex. The development cost of a clinical drug project can exceed 350 million US dollars. If marketing expenses are included, the total cost can exceed one billion US dollars [182]. Bone-targeting nanoparticles, as an emerging and cutting-edge treatment approach, are expected to be more expensive. On the one hand, nanoparticles require high-quality raw materials, and many developing countries need to import a large amount of raw materials. On the other hand, the stability of nanomaterials is relatively poor compared to other dosage forms and requires strict storage conditions, which further increases the therapeutic burden of nanomaterials. The production of nanoparticles involves multiple technical processes, and the risk of patent infringement also deters many companies [183]. According to statistics, the average treatment cost of nanopreparations is several times that of traditional preparations, which is a considerable burden for patients, especially those in low- and middle-income countries.

### 3.12. Future Development Trends

Future research on bone-targeting nanoparticles will no longer be confined to simple “delivery”, but will move towards greater intelligence, greater collaboration, and integrated diagnosis and treatment.

#### 3.12.1. Multi-Target or Intelligent Responsive Nanoparticles

Although bone-targeting nanoparticles can be directed to bone tissue, the issue of random distribution within the bone throughout the body remains [184]. Therefore, secondary targets or intelligent, responsive nanoparticles targeting lesion areas are crucial for enhancing the specificity and effectiveness of bone-targeting nanoparticles. Their core value lies in utilizing specific signals from the bone pathological microenvironment or external controllable stimuli to achieve precise spatiotemporal delivery and regulation of drug action, providing advanced, additional, and alternative options of therapy [185]. The characteristic microenvironment of osteoporotic lesion areas provides natural signals for nanoparticles. pH-responsive, ROS-responsive, and enzyme-responsive bone-targeting nanoparticles are expected to receive more research in the future.

#### 3.12.2. Multi-Mechanism Synergistic Treatment

Bone-targeting nanoparticles for multi-mechanism synergistic treatment exert therapeutic effects by integrating multiple strategies such as anti-bone resorption, promotion of bone formation, immune regulation, and modulation of the gut microbiota. They fully leverage the advantages of advanced treatment technologies, specifically address the complex pathological mechanisms of osteoporosis, overcome the limitations of single therapies, and provide technical support for the efficient treatment of osteoporosis. Shunyi Lu et al. creatively prepared a magnesium gelatin composite microsphere scaffold. When the scaffold entered the implantation site, body fluids entered the cavity of the microporous structure, and a chemical reaction was produced to generate anti-inflammatory hydrogen gas. In addition, the acidic microenvironment of the osteoporotic lesion site accelerated this reaction. It fully exploits the characteristics of hydrogen, such as its rapid diffusion speed and wide distribution range. This scaffold simultaneously achieved dual-mechanism synergistic therapy, inhibiting pyroptosis and providing hydrogen therapy. The results showed that it reduced the expression level of CD86, a marker of M1-type macrophages, in LPS-treated samples from 6.24% to 4.76%, and increased the expression level of CD206, a marker of M2-type macrophages, from 0.23% to 33.2%. In the OVX mouse skull defect model, the implantation of this nanoscaffold significantly increased bone density and trabecular bone count, demonstrating strong potential for anti-osteoporosis [186]. There has been a fundamental breakthrough in the treatment of osteoporosis treatment, shifting from a “multi-dimensional synergy” to the innovative potential of bone-targeted nanotechnology.

## 4. Conclusions

Osteoporosis has become a widespread issue affecting countries around the globe. This review summarizes the commonly used clinical anti-osteoporosis drugs, the raw materials, preparation methods, and the action mechanisms of bone-targeting nanoparticles. Osteoporosis involves various pathophysiological mechanisms, and the currently available anti-osteoporosis medications often come with side effects and other complications that can significantly harm patients’ health. Bone-targeting nanoparticles achieve bone tissue enrichment by incorporating bone-affinity molecules. With the core of regulating the balance between bone resorption and bone formation, these nanoparticles overcome the limitations of traditional therapies, such as low specificity and significant side effects, improve patients’ medication compliance, and significantly enhance therapeutic effects. However, there are also some challenges in the clinical translation of bone-targeted nanoparticles which require further investigation. Bone-targeting nanoparticles are expected to advance significantly through future research, leading to safer, more effective, and more convenient treatment options for patients with osteoporosis.

## Figures and Tables

**Table 1:** Terms used in the search strategy.

Electronic Database	Search and Terms
Web of Science PubMed	#1((Osteoblast) OR (Osteoclast)) OR (Osteoporosis) #2 (nanoparticles) AND (bone)
	#3 ((((((((Bisphosphonates) OR (Alendronic)) OR (Zoledronic)) OR (Teriparatide)) OR (Osteoporosis)) OR (Anabolic Agents)) OR (Anti-Resorptive Agents)) OR (Abaloparatide)) AND (Osteoporosis) #4((((targeted) OR (targeting)) OR (responsive)) AND (((Nanoparticles) OR (Nanometer particle)) OR (Nano formulation))) AND (bone)

**Table 2:** The effects and mechanisms of anti-osteoporosis drugs.

Drug Name	Drug Category	Mechanism of Action	Effect and Function	Site of Action ^a^	Effect Size	Reference
Bisphosphonates	bisphosphonates	Anti-Resorptive	Increase bone density	LS	6.8%↑	[40]
Alendronate	Bisphosphonates	Anti-Resorptive	Increase bone density	LS, TH, TR	3.7%↑, 2.1%↑, 1.7↑%	[41]
Alendronate	Bisphosphonates	Anti-Resorptive	Increase bone density	LS, TH, FN	5.2%↑, 2.3%↑, 2.5%↑	[42]
Zoledronic acid	Bisphosphonates	Anti-Resorptive	Increase bone density, etc.	LS, TH, FN	6.1%↑, 3.1%↑, 3.9%↑	[43]
Zoledronic acid	Bisphosphonates	Anti-Resorptive	Increase bone density, etc.	LS, TH	7.1%↑, 4.4%↑	[44]
Zoledronic acid	Bisphosphonates	Anti-Resorptive	Increase bone density, etc.	LS, TH, FN	6.1%↑, 3.8%↑, 3.1%↑	[42]
Denosumab	RANKL inhibitor	Anti-Resorptive	Increase bone density, etc.	LS	7.7%↑	[45]
Denosumab	RANKL inhibitor	Anti-Resorptive	Increase bone density, etc.	LS	7.2%↑	[46]
Denosumab	RANKL inhibitor	Anti-Resorptive	Increase bone density, etc.	LS, TH	9.7%↑, 5.1%↑	[44]
Denosumab	RANKL inhibitor	Anti-Resorptive	Increase bone density, etc.	LS, TH, FN	7.3%↑, 3.6%↑, 3.2%↑	[47]
Denosumab	RANKL inhibitor	Anti-Resorptive	Increase bone density	LS, TH, FN	5.8%↑, 2.3%↑, 2.1%↑	[42]
Teriparatide	PTH	Anabolic Agents	Increase bone density, etc.	LS	12.0%↑	[40]
Teriparatide	PTH	Anabolic Agents	Reduce the risk of fractures	HF	56.0%↓	[48]
Teriparatide	PTH	Anabolic Agents	Reduce the risk of fractures	VF	80.0%↓	[49]
Teriparatide	PTH	Anabolic Agents	Increase bone density	LS	5.5%↑	[50]
Teriparatide	PTH	Anabolic Agents	Reduce the risk of fractures	NVF	53.0%↓(T2D) 43.0%↓(non-T2D)	[51]
Teriparatide	PTH	Anabolic Agents	Increase bone density	LS, FN	8.2%↑, 1.3%↑	[42]
Abaloparatide	PTH	Anabolic Agents	Reduce the risk of fractures	VF, NVF	86.0%↓, 43.0%↓	[49]
Abaloparatide	PTH	Anabolic Agents	Increase bone density	LS, TH, FN	11.3%↑, 3.9%↑,4.0%↑	[42]
Romosozumab	Monoclonal antibody	Dual effect	Increase bone density	LS, TH, FN	12.1%↑, 2.5%↑, 2.2%↑	[42]

^a^ LS: Lumbar Spine; TH: Thoracic Spine; TR: Trochanter; FN: Femoral Neck; HF: Hip fractures; VF: Vertebral Fracture; NVF: Non-Vertebral Fracture.

**Table 3:** Properties of bone-targeted nanoparticles.

Carrier Material	Targeted Ligand	Other Substance	Target Type	Targeted Verification Method	Effect and Function	Reference
PLGA, CS, CD	Alendronate	-	Active targeting	HAP affinity	Continuously release drugs and precisely target the bone matrix	[159]
PLGA-TK-PEG, BMSCM	DSPE-PEG-ALN	SS-31	Active targeting	Biological distribution	Antioxidant stress	[135]
PLGA	Tetracycline	Astragaloside A Icariin Notoginsenoside R1	Active targeting	HAP affinity, biological distribution	Osteoblast proliferation and differentiation	[76]
PEG-PLGA	(Aspartic acid)3	Simvastatin	Active targeting	Biological distribution	Osteoblast proliferation and differentiation	[113]
PLGA	HMEC membranes overexpressing CXCR4	Sec	Active targeting	Biological distribution	Proliferation and differentiation of osteoblasts, inhibition of osteoclasts	[160]
PLGA	Alendronate	Osteogenic peptide	Active targeting	Biological distribution, HAP affinity	Osteoblast proliferation and differentiation	[161]
Lipids, PLGA	APT, Alendronate	SFRP1 silent GapmeR	Active targeting	-	Osteoblast proliferation and differentiation	[162]
Chitosan	Asp-8	Cyclic peptide J	Active targeting	Biological distribution, HAP affinity	Osteoblast proliferation and differentiation	[80]
Polymer micelles	Alendronate	Icaritin	Active targeting	Biological distribution, HAP affinity	Osteoblast proliferation and differentiation	[105]
Polyurethane (PU) nano-micelles	Bone-targeted peptide (SDSSD)	SiRNA/miRNA	Active targeting	Biological distribution	RNAi therapy	[163]
Polymer micelles	Citric acid	Estrogen	Active targeting	Biological distribution, HAP affinity	Inhibit osteoclasts	[164]
Liposome	Adapter	SiRNA	Active targeting	Biological distribution	Osteoblast proliferation and differentiation	[87]
Liposome	Alendronate	Pomolic acid	Active targeting	Biological distribution	Inhibit osteoclasts	[165]
Liposome	Bone-targeted peptide (SDSSD)	PTH (1–34)	Active targeting	HAP affinity	Targeted drug delivery	[166]
Liposome	Asp-8	PTX	Active targeting	Biological distribution, HAP affinity	Targeted drug delivery	[109]
Liposome	Bone affinity peptide (DSS)_6_	Quercetin	Active targeting	Biological distribution	Eliminate senescent cells, etc.	[167]
Hydroxyapatite	Alendronate sodium	Tanshinol	Active targeting	Biological distribution	Targeted drug delivery	[168]
Zinc hydroxyapatite	Risedronate	-	Active targeting	-	Provide trace elements	[169]
Hydroxyapatite	-	salmon calcitonin	Passive targeting	-	Targeted drug delivery	[98]
Cerium oxide	Alendronate sodium	-	Active targeting	-	Inhibition of osteoclasts	[170]
Ferritin	Asp-6	-	Active targeting	Biological distribution, HAP affinity	Bone imaging	[171]
ZIF-8, PVP	Zoledronate	DOX, BSA, SiRNA	Active targeting	Biological distribution	Targeted drug delivery	[88]
Paramagnetic iron oxide	(-D-Asp-)8	-	Active targeting	Biological distribution	Diagnosis of osteoporosis	[172]
Gold	Alendronate	-	Active targeting	Biological distribution	Osteoblast proliferation and differentiation	[173]
CM-NH2-PAA	Alendronate sodium	-	Active targeting	Biological distribution, HAP affinity	Deliver trace elements and resist oxidative stress	[122]
Mesoporous silica	Alendronate	SiRNA, Osteostatin	Active targeting	HAP affinity	Proliferation and differentiation of osteoblasts, inhibition of osteoclasts	[100]
Exosome mimetics	Alendronate	-	Active targeting	Biological distribution, HAP affinity	Cell adhesion, etc.	[174]
mEVs	(AspSerSer, DSS)_6_	SRT2104	Active targeting	Biological distribution	Proliferation and differentiation of osteoblasts, inhibition of osteoclasts	[111]
mEVs	(AspSerSer, DSS)_6_	Antagomir-483-5p	Active targeting	-	Promote osteogenic differentiation, etc.	[175]
CCMV	Peptide RM	-	Active targeting		Inhibition of osteoclasts	[176]
mSiO_2_, β-NaYF4	EDTA	α-ketoglutarate	Active targeting	Biological distribution	Proliferation and differentiation of osteoblasts, inhibition of osteoclasts	[177]

## Data Availability

No new data were created or analyzed in this study. Data sharing is not applicable to this article.

## Supplementary Materials

The following supporting information can be downloaded at: https://www.mdpi.com/article/10.3390/ph18121809/s1, PRISMA 2020 Checklist.

## Abbreviations

The following abbreviations are used in this manuscript:

ALP	Alkaline phosphatase
PDGF	Platelet-Derived Growth Factor
BMPs	Bone Morphogenetic Proteins
IGFs	Insulin-Like Growth Factors
RANKL	Receptor Activator of Nuclear Factor-Kappa B Ligand
MOF	major osteoporotic fractures
PTH	Parathyroid hormone
LS	Lumbar Spine
TH	Thoracic Spine
TR	Trochanter
FN	Femoral Neck
Hip	Hip Joint
VF	Vertebral Fracture
NVF	Non-Vertebral Fracture
HF	Hip fractures
PAD	Pamam dendrimer
HCCP	Hexachlorocyclotriphosphazene
Cur	Curcumin
PLGA	Polylactic-glycolic acid copolymer
NPs	Nanoparticles
CCK-8	Cell Counting Kit-8
Ovariectomized	OXV
OXV	Ovariectomized
CS	Chitosan
PEG	Polyethylene Glycol
BMD	Bone mineral density
BV/TV	Bone volume fraction
MOF	Metal–organic framework
Tb.N	Trabecular bone count
AuNPs	Gold nanoparticles
MSC	Mesenchymal stem cells
HAP	Hydroxyapatite
HANP	Hydroxyapatite nanoparticles
SCT	Salmon calcitonin
TC	Tetracycline
Asp	Aspartic Acid
Ser	Serine
Gly	Glycine
Arg	Arginine
DSPE	1,2-Distearoyl-sn-glycero-3-phosphoethanolamine
MAL	Maleimide
ROS	Reactive oxygen species
BMMs	Bone marrow-derived macrophages
CD	Cyclodextrin
mEVs	Milk-derived extracellular vesicles
CCMV	Cowpea chlorotic mottle virus
EDTA	Ethylene diamine tetraacetic acid
